# Investigation of the introduction of three times daily injections of Insulin Lispro Mixture-50 on an outpatient basis: Therapeutic effects of 12 months’ treatment with and without concomitant sulfonylurea

**DOI:** 10.1111/j.1753-0407.2012.00190.x

**Published:** 2012-09

**Authors:** Katsunori Suzuki, Hiroshi Morikawa, Midori Iwanaga, Yoshifusa Aizawa

**Affiliations:** 1 Division of Endocrinology and Metabolism, Saiseikai Niigata Second Hospital; 2 Division of Endocrinology and Metabolism, First Department of Internal Medicine, Niigata University School of Medicine, Niigata, Japan

**Keywords:** Insulin Lispro Mixture, outpatient, sulfonylureas, type 2 diabetes

There is no definitive evidence as to whether oral hypoglycemic agents (OHA) should be continued or withdrawn prior to the introduction of insulin therapy on an outpatient basis. Insulin Lispro Mixture-50 (LM-50), which supplements basal insulin secretion because of the presence of the intermediate-acting component contained in it, may enable more stable glucose control and allow discontinuation of prolonged OHA therapy, such as with sulfonylureas (SU). To determine whether the introduction of three times daily (t.i.d.) injections of LM-50 on an outpatient basis was effective in yielding adequate glucose control in patients with type 2 diabetes (T2D) who had poor glucose control despite treatment with OHA, patients were divided into two groups: those in whom the use of all OHA except SU was suspended [the SU(+) group; *n* = 10] and those in whom all OHA, including SU, were discontinued [the SU(−) group; *n* = 12].

Twenty-two patients with T2D undergoing treatment at Saiseikai Niigata Second Hospital were included in the study. Patients had been treated with maximum doses of OHA, but had failed to show adequate glucose control (HbA1c ≥7%). All patients refused inpatient therapy, but agreed to receive LM-50 injections t.i.d. on an outpatient basis. All patients provided informed consent for inclusion in the present study, which was an open-label, randomized, active-controlled, parallel-group, comparative study. For patients in the SU(+) group, SU was continued at the same dose and LM-50 injections were started at a dose of 0.1 units/kg, t.i.d. Patients in the SU(−) group were started on the same dose of LM-50, but all OHAs were discontinued. The primary efficacy endpoint was the change in HbA1c level.

After 1–2 months of LM-50 injections, patients began to get used to the self-monitoring of blood glucose levels and the dose of LM-50 injected was adjusted with the aim of reducing preprandial blood glucose levels to ≤130 mg/dL.

Changes in HbA1c levels for patients in both the SU(+) and SU(−) groups are shown in Fig. 1. For the 10 patients in the SU(+) group, significant decreases in HbAlc started to be seen after 1 months treatment with LM-50. Similarly, patients in the SU(−) group exhibited significant decreases after 1 months treatment with LM-50. At the end of 12 months, significant improvements were observed in both the SU(+) and SU(−) groups, with the effects of LM-50 t.i.d. almost the same, regardless of whether SU was continued or discontinued. In the SU(+) group, the percentage of patients in whom HbA1c fell to <7.0% or <6.5% was 50% and 20%, respectively, compared with 66.7% and 41.7%, respectively, in the SU(−) group. There was no significant difference in the percentage change in HbA1c or the percentage of patients in whom HbA1c fell to <7.0% and <6.5% between the two groups.

**Figure 1:**
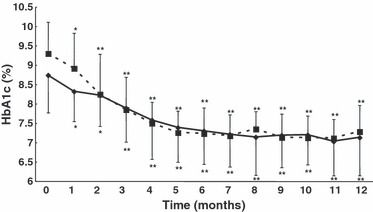
Mean HbA1c levels in patients in whom the use of all oral hypoglycemic agents (OHA), except sulfonylureas (SU), was suspended [SU(+) group; *n* = 10; ◆] and those in whom all OHA, including SU, were discontinued [SU(−) group; *n* = 12; 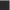]. The decrease in HbA1c levels at each time point was significantly different compared with baseline levels (Month 0) in both groups. Data are the mean ± SD. *P < 0.05, **P < 0.01 compared with baseline.

The body mass index (BMI) and insulin dose administered increased significantly in both the SU(+) and SU(−) groups over the 12-month treatment period (Table 1). There were no significant differences in terms of the percentage change in BMI or the insulin dose between the two groups (Table 1).

**Table 1:** Baseline characteristics and the outcomes and changes from baseline after 12 months treatment of patients in whom the use of all oral hypoglycemic agents (OHA), except sulfonylureas (SU), was suspended [SU(+) group] and those in whom all OHA, including SU, were discontinued [SU(−) group]

	Total (*n* = 22)	SU(+) group (*n* = 10)	SU(−) group (*n* = 12)	*P*-value^†^
Sex (M/F)	14/8	8/2	6/6	
Age (years)	56.5 ± 9.3	54.7 ± 10.5	58.0 ± 8.3	0.21
Duration of diabetes (years)	13.1 ± 7.0	13.9 ± 7.1	12.4 ± 7.1	0.31
Baseline				
HbA1c (%)	9.0 ± 1.0	8.7 ± 0.8	9.3 ± 1.0	0.08
BMI (kg/m^2^)	25.7 ± 5.9	28.2 ± 6.7	23.7 ± 4.5	0.05
Insulin dose (U/kg)	0.23 ± 0.18	0.21 ± 0.05	0.30 ± 0.05	<0.01
After 12 months treatment				
HbA1c (%)	7.2 ± 0.8**	7.2 ± 0.7**	7.3 ± 1.0**	0.39
Δ HbA1c from baseline (%)	−1.80 ± 1.07	−1.55 ± 0.97	−2.02 ± 1.14	0.16
% Change in HbA1c from baseline	−19.44 ± 10.33	−17.26 ± 9.68	−21.27 ± 10.92	0.19
BMI (kg/m^2^)	27.1 ± 5.4**	29.3 ± 5.7*	25.3 ± 4.5**	0.05
% Change in BMI from baseline	5.9 ± 4.3	4.5 ± 4.0*	7.0 ± 4.3	0.08
Δ Insulin dose from baseline (U/kg)	0.49 ± 0.16**	0.47 ± 0.12**	0.52 ± 0.19**	0.23
% Patients with HbA1c <7.0%	59.1	50.0	66.7	0.22
% Patients with HbA1c <6.5%	31.8	20.0	41.7	0.14

Unless indicated otherwise, data are given as the mean ± SD.

* *P *< 0.05, **P < 0.01 compared with baseline; ^†^refers to comparison of SU(+) group with SU(−) group.

BMI, body mass index.

Furthermore, although the incidence of slight hypoglycemia was the same in the SU(+) and SU(−) groups, serious hypoglycemia did not occur in any of the patients in either group during the 12-month observation period.

In general, it is believed that when insulin therapy is introduced on an outpatient basis, complete discontinuation of SU treatment often results in a decrease in the amount of insulin that enters the portal vein, thereby exacerbating hyperglycemia. However, this scenario was not observed in the present study, probably because the LM-50 used in the present study contained both an ultraquick-acting component and an intermediate-acting component (in a ratio of 1:1) and the intermediate-acting component acted in lieu of the basal secretory component induced by SU treatment. Because the LM-50 used in the present study contains bolus insulin and basal insulin at a ratio of 1:1, it is believed that this provides insulin in a pattern that approximates physiological secretion in humans.^1^,^2^

In conclusion, LM-50 injections administered three times daily produced almost the same effects in patients with initially poor glycemic control on OAH, regardless of whether SU was continued.

## Disclosure

The authors declare that there are no conflicts of financial interest in terms of the information contained in this paper.
